# Association Between Long-term Weight Change Since Midlife and Risk of Incident Disabling Dementia Among Elderly Japanese: The Ohsaki Cohort 2006 Study

**DOI:** 10.2188/jea.JE20200260

**Published:** 2022-05-05

**Authors:** Yukai Lu, Yumi Sugawara, Sanae Matsuyama, Ichiro Tsuji

**Affiliations:** Division of Epidemiology, Department of Health Informatics and Public Health, Tohoku University School of Public Health, Graduate School of Medicine, Sendai, Japan

**Keywords:** weight loss, weight change, dementia, cognition, prospective cohort study

## Abstract

**Background:**

Both weight loss and cognitive impairment are common in late-life, but it remains unknown whether weight change is associated with risk of incident dementia among elderly Japanese. Our study aimed to investigate the association between long-term weight change since midlife and risk of incident disabling dementia using a community-based cohort study of elderly Japanese.

**Methods:**

In 2006, we conducted a cohort study of 6,672 disability-free Japanese adults aged ≥65 years. In both 1994 and 2006, the participants reported their weight using a self-reported questionnaire. Based on weight obtained at these two time points, participants were classified into: stable weight (−1.4 to +1.4 kg), weight gain (≥+1.5 kg), and weight loss of −2.4 to −1.5 kg, −3.4 to −2.5 kg, −4.4 to −3.5 kg, −5.4 to −4.5 kg, and ≥−5.5 kg. Incident disabling dementia was retrieved from the public Long-term Care Insurance database. Participants were followed-up for 5.7 years (between April 2007 and November 2012). Cox proportional hazards model was used to estimate multivariable-adjusted hazard ratios (HRs) and 95% confidence intervals (CIs) for incident disabling dementia.

**Results:**

During 32,865 person-years of follow-up, 564 participants were ascertained as having incident disabling dementia. Compared with stable weight, the multivariable-adjusted HRs were 0.97 (95% CI, 0.70–1.34) for weight loss of −2.4 to −1.5 kg, 0.98 (95% CI, 0.70–1.38) for −3.4 to −2.5 kg, 1.28 (95% CI, 0.91–1.81) for −4.4 to −3.5 kg, 1.27 (95% CI, 0.92–1.77) for −5.4 to −4.5 kg, and 1.64 (95% CI, 1.29–2.09) for ≥−5.5 kg.

**Conclusion:**

Our study suggested that a ≥−3.5 kg weight loss over 12 years might be associated with higher risk of incident disabling dementia among elderly Japanese.

## INTRODUCTION

The number of people living with dementia is rising rapidly worldwide, estimated at 47 million in 2015 and projected to triple by 2050.^1^ Although the incidence rate of dementia has been reported to have declined in Europe and North America,^2^ it is still increasing in most countries worldwide.^1^ Thus, it is crucial to identify modifiable risk factors for preventing dementia.

Obesity in midlife is an important risk factor for incident dementia,^3^ and being underweight in late-life also seems to be associated with higher risk of incident dementia.^4^ There has been accumulating evidence that weight loss during adulthood is associated with dementia.^5^^–^^13^ However, most studies were conducted among Caucasians,^7^^–^^13^ whom usually have higher body weight and weight change than Asians. One study suggested that a weight loss of ≤−7.5 kg over 30 years was significantly associated with risk of death from dementia,^7^ whereas others reported a >5% loss in body mass index (BMI) over 3 to 9 years was associated with dementia risk.^8^^,^^12^ Only one study was conducted in Asia, which was a report from South Korea that also found a >10% loss in BMI over a 2-year period was associated with dementia.^6^ However, it remained unstudied whether body weight loss had an association with dementia among Japanese elderly. We hypothesized that weight loss might be consistently associated with dementia among Japanese elderly, but a smaller amount of weight loss would have an association with an elevated risk.

Thus, our study aimed to examine the association between long-term weight change since midlife and risk of incident disabling dementia, with particular focus on exploring the specific amount of weight loss at which people started showing an elevated risk, among elderly Japanese using a community-based cohort study.

## METHODS

### Study cohort

Similar to our previous studies,^14^^,^^15^ we used merged data from the Ohsaki Cohort Study and the Ohsaki Cohort 2006 Study, details of which have already been described elsewhere.^16^^,^^17^

The Ohsaki Cohort 2006 Study was a community-based cohort study conducted in Ohsaki City, Miyagi Prefecture, Japan. All the citizens of Ohsaki City aged ≥65 years were invited to participant in the baseline survey, which was conducted from December 1–15, 2006. Then, the participants were followed-up from April 1, 2007.

In the same area, a health-related questionnaire survey (the 1994 Survey) was also conducted between October and December 1994. Although only National Health Insurance beneficiaries were invited in the 1994 Survey, some citizens of Ohsaki city participated in both surveys.

**Figure 1.**
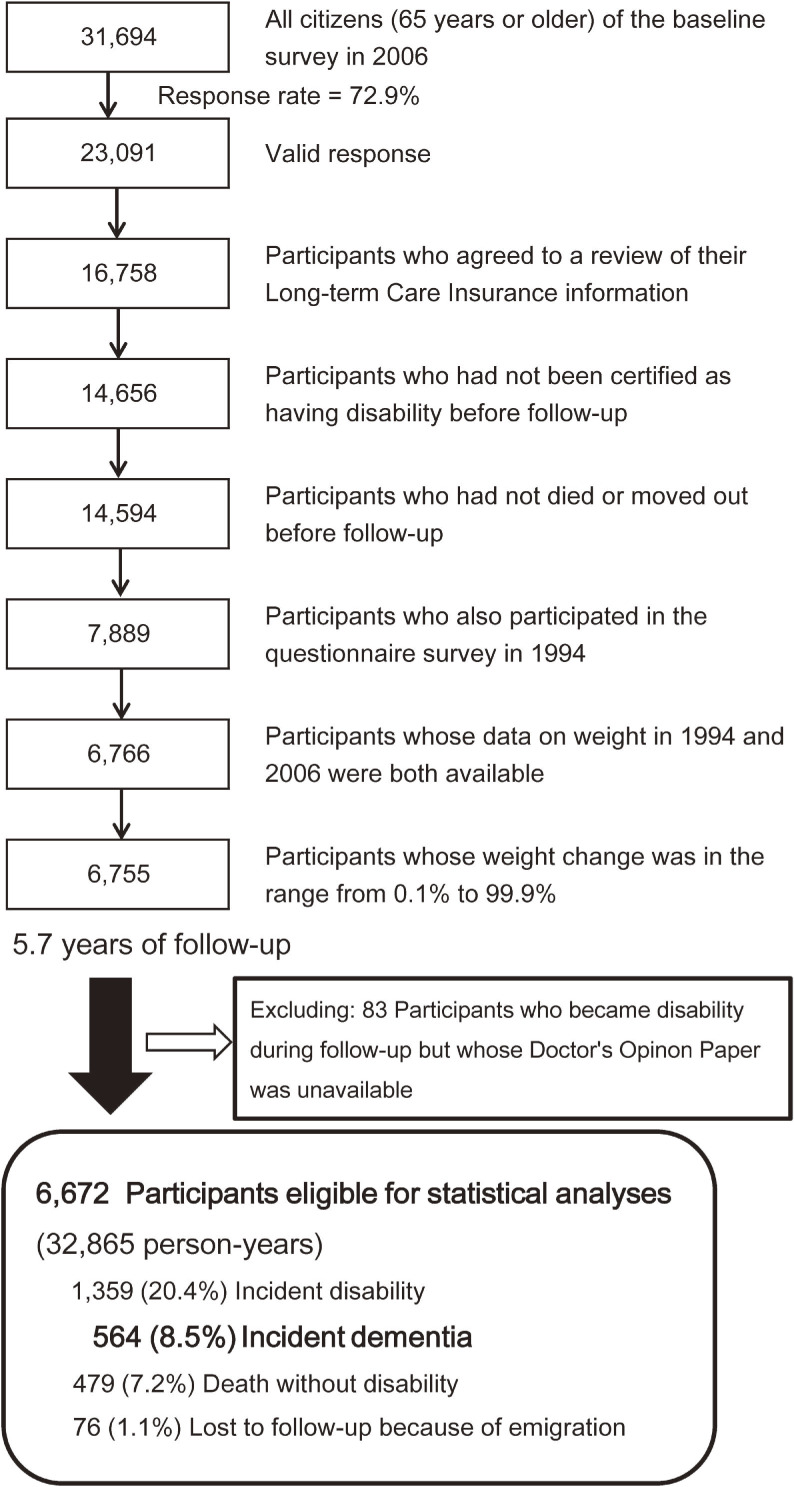
Flow chart of study participants

At baseline, 23,091 out of 31,694 eligible participants (72.9%) provided valid responses and formed the cohort study. We then excluded participants who disagreed to a review of their Long-term Care Insurance (LTCI) information, who had already been certified as having disability by the LTCI or had died or emigrated before follow-up, who had not participated in the 1994 Survey, whose data on weight in either 1994 or 2006 were missing, and whose amount of weight change was out of the 0.1–99.9% distribution. Thereafter, we also excluded participants certified as having disability during follow-up who were lack of their Doctor’s Opinion Paper (DOP). The DOP is a standard form used for assessing patients’ chronic medical conditions and functions of daily life. Finally, 6,672 participants were included for the statistical analysis (Figure 1).

### Weight change (exposure)

Data on self-reported weight were collected from the baseline survey and the 1994 Survey. The amount of weight change was calculated as self-reported weight in 2006 subtracted by self-reported weight in 1994. Thereafter, participants were primarily classified into three groups, a weight loss (≤−1.5 kg) group, a stable weight (−1.4 to +1.4 kg) group, and a weight gain (≥+1.5 kg) group. We further divided people with weight loss into five groups of −2.4 to −1.5 kg, −3.4 to −2.5 kg, −4.4 to −3.5 kg, −5.4 to −4.5 kg, and ≥−5.5 kg.

### Follow-up (incident disabling dementia)

Incident disabling dementia was defined as the primary outcome according to the criteria of the LTCI system that has been implemented in Japan since April 2000.^18^ The LTCI is a mandatory form of national social insurance to assist activities of daily living in the disabled elderly provided by municipalities.^19^ People aged ≥40 years paying premiums and those aged ≥65 years are eligible to apply for formal caregiving services. People were ascertained as having disability under a uniform standard of disability certification which consist of two parts of information source: one is assessment of the degree of functional disability using a standardized questionnaire developed by the Minister of Health, Labour and Welfare, and the other is reference to the DOP prepared by the attending physician.

Thereafter, among people ascertained as having disability, we defined disabling dementia as exceeding rank I (ie, ≥rank II) on the Dementia Scale (Degree of Independence in Daily Living for Elderly with Dementia), as entered on the DOP. This cutoff point for defining incident disabling dementia has been used in previous studies.^18^^,^^20^ The Dementia Scale were reported to have a satisfying sensitivity and specificity against clinical diagnoses by neuropsychiatrists.^21^

All participants were followed up by reviewing information on LTCI certification, death, or emigration from Ohsaki City. All data were transferred annually in December from the Ohsaki City Government under an agreement related to Epidemiologic Research and Privacy Protection.

### Covariates

We obtained information on socio-demographic, lifestyle, and health-related variables in both 1994 and 2006. Body mass index (BMI) was calculated as the self-reported weight (kg) divided by the square of the self-reported height (m). Energy intake was obtained based on a 39-item food frequency questionnaire which has been validated previously,^15^^,^^22^ and changes in energy intake between 1994 and 2006 were calculated. Persistently walking for ≥1 h/d was defined as reporting ≥1 h/d of walking in both 1994 and 2006. Any new reported disease was defined as reporting history of diseases in 2006 but not in 1994. In 2006, psychological distress was additionally measured using the Kessler 6-item Psychological Distress Scale.^23^^,^^24^ We classified individuals with a score ≥13 as having psychological distress.^23^

### Ethical issues

Informed consent was obtained from all individual participants included in the study. We considered the return of completed questionnaires to imply consent to participate in the study involving surveys data and subsequent follow-up of death and emigration. We also confirmed information regarding LTCI certification status after obtaining written consent along with the questionnaires returned from subjects at the time of the baseline survey in 2006. The Ethics Committee of Tohoku University Graduate School of Medicine (Sendai, Japan) reviewed and approved the study protocol (approval Nos. 2014-1-839 and 2016-1-586).

### Statistical analysis

We counted the person-years of follow-up for each subject from April 1, 2007 until the date of ascertainment of incident disabling dementia, date of emigration from the study area, date of death, date of incident disability without dementia or the end of the follow-up (November 30, 2012), whichever occurred first.

Kaplan-Meier curves of dementia-free probability distribution were drawn, and log-rank tests were conducted using the multiple comparison method (ie, Dunnett’s test) to examine differences between the reference group and the other groups.

The multivariable-adjusted Cox proportional hazards model was used to calculate the hazard ratios (HRs) and 95% confidence intervals (CIs) for incident disabling dementia according to weight change with “stable weight” as the reference category. Dummy variables were created for each group of the exposure and covariates. Multivariable models were adjusted for the following variables. Model 1 was adjusted for sex and age (continuous); model 2 was further adjusted for education level, smoking status, time spent walking, psychological distress, history of diseases in 2006, and BMI in 1994; and model 3 was further adjusted for changes in covariates between 1994 and 2006. To test for linear trends, categories indicating weight loss were entered as a continuous variable in corresponding models.

Then, we also conducted stratification analysis according to sex, age in 1994 (<60 or ≥60 years), and BMI in 1994 (<25.0 or ≥25.0 kg/m^2^) to investigate any differences existing in the association between weight change and dementia. In addition, tests of interaction between weight loss and stratified variables were also performed by adding a cross-product term to corresponding models. An unintentional weight loss of ≤−2 to −3 kg/6 months was one of the indicators when evaluating frailty among Japanese elderly.^25^^,^^26^ We also conducted a sensitivity analysis by excluding 1,224 participants who reported an unintentional weight loss of ≤−2 to −3 kg/6 months at baseline using the Kihon Checklist, attempting to minimize the effect of short-term weight loss due to frailty.

Kaplan-Meier curves were drawn by STATA/MP 16.1 (StataCorp, College Station, TX, USA). All the other analyses were performed using SAS version 9.4 (SAS Institute, Cary, NC, USA). All statistical tests described here were two-sided, and differences at *P* < 0.05 were accepted as statistically significant.

## RESULTS

During 32,865 person-years of follow-up, among 6,672 participants (mean age, 74.5; standard deviation [SD], 5.5 years old; male: 43.8%), 564 (8.5%) participants were ascertained as having incident disabling dementia. Table 1 shows the characteristics of the participants according to categories of weight change. Participants with weight loss were more likely to be older, smokers, have psychological distress, have a history of MI, diabetes, or cancer, and have decreased daily energy intake (Table 1).

**Table 1.** Characteristics of participants according to weight change (*n* = 6,672)

	Weight change			*P*-value^f^

	Weight loss	Stable weight	Weight gain	
	(≤−1.5 kg)	(−1.4 to +1.4 kg)	(≥+1.5 kg)	
Number of participants	3,113	1,949	1,610	
Weight changes, kg, mean (SD)	−5.2 (3.4)	0.0 (0.7)	4.4 (2.8)	<0.0001
Age, yr., mean (SD)	75.4 (5.6)	73.9 (5.2)	73.6 (5.4)	<0.0001
Male, %	43.5	41.7	46.8	0.0086
Junior high school or lower, %^a^	31.5	28.3	30.8	0.2033
Current smokers, %	14.9	12.0	12.5	<0.0001
Time spent walking (<0.5 h/day), %	35.3	31.8	36.1	0.0031
Psychological distress, %^b^	6.0	3.4	4.8	0.0008
History of diseases, %				
Stroke	2.8	2.5	3.5	0.1792
Hypertension	54.5	56.6	62.1	<0.0001
Myocardial infarction	5.8	3.0	4.9	<0.0001
Gastric and duodenal ulcer	16.5	14.6	15.3	0.2047
Diabetes	14.0	9.0	10.1	<0.0001
Cancers	10.7	5.9	6.9	<0.0001
BMI in 1994, kg/m^2^, mean (SD)	23.9 (3.0)	23.5 (2.9)	23.4 (2.9)	<0.0001
BMI in 2006, kg/m^2^, mean (SD)	22.3 (3.1)	23.8 (3.0)	25.5 (3.2)	<0.0001
Changes in energy intake per day, kcal, mean (SD)^c^	−81.6 (525.9)	−35.8 (540.1)	−34.6 (509.8)	0.0016
Persistently walking for ≥1 h/day, %^d^	19.1	23.5	18.5	0.0003
Any new reported disease, %^e^	40.8	34.3	37.9	<0.0001

BMI, body mass index.

^a^Age when completing education ≤16 y.

^b^Kessler 6-item Psychological Distress Scale score ≥13.

^c^Changes in daily energy intake = daily energy intake in 2006 − daily energy in take in 1994.

^d^Participants reporting time spent walking for 1 h/day in both 1994 and 2006.

^e^Participants reporting history of stroke, hypertension, myocardial infarction, gastric and duodenal ulcer, diabetes, or cancers in 2006 not in 1994.

^f^Calculated by ANOVA test for continuous variables and χ2 test for categorical variables.

**Figure 2.**
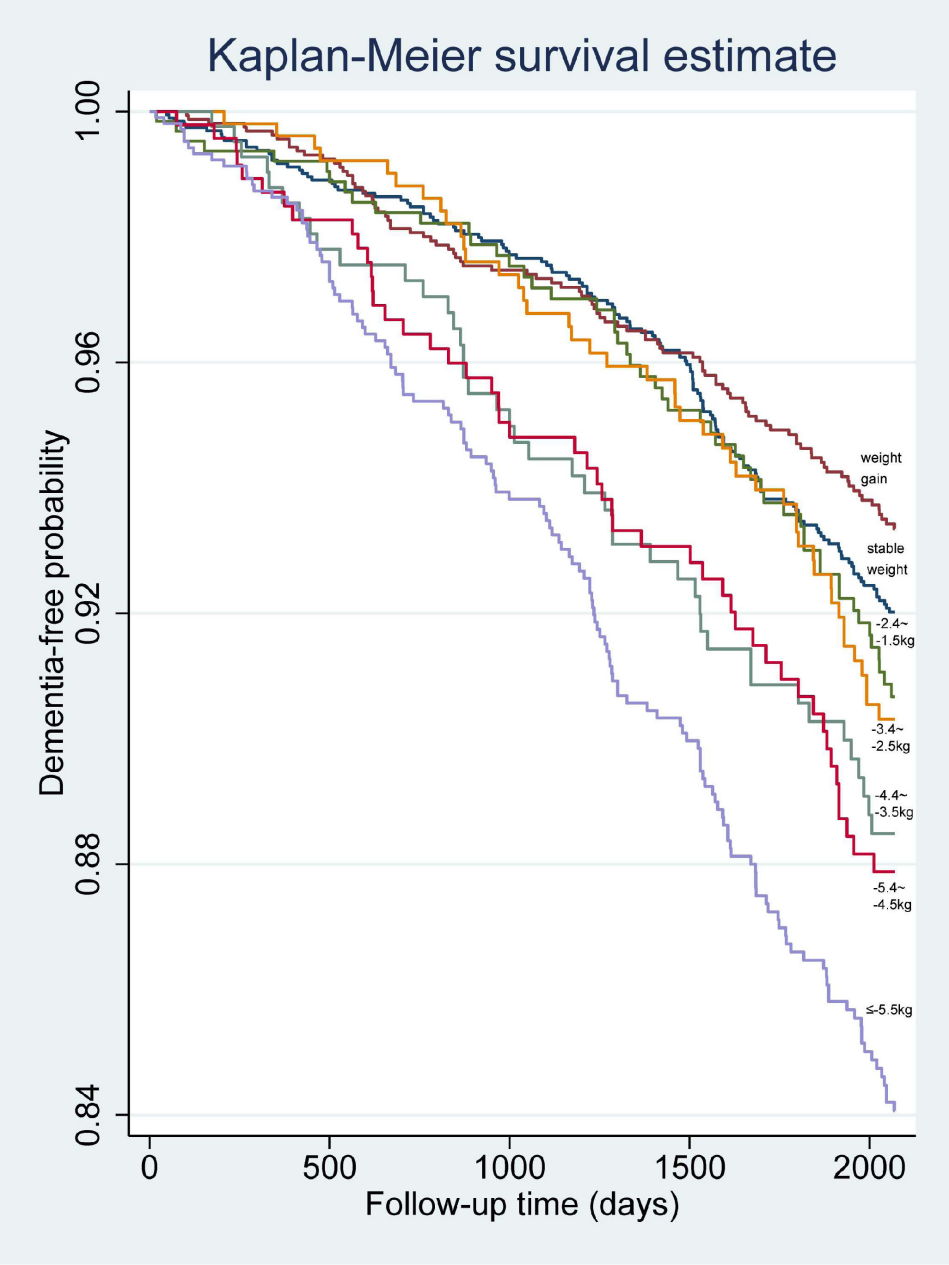
Kaplan–Meier curves of dementia-free probability distribution according to weight change groups

Figure 2 shows the Kaplan-Meier curves of dementia-free probability distribution. The results of the log-rank tests suggested that compared with stable weight, groups of −4.4 to −3.5 kg, −5.4 to −4.5 kg, and ≤−5.5 kg of weight loss showed a significant difference (data not shown).

Table 2 and Table 3 shows the association between weight change and risk of incident disabling dementia. Compared to participants with stable weight, the HRs were 1.26 (95% CI, 1.03–1.54) for weight loss and 0.82 (95% CI, 0.63–1.06) for weight gain. The results remained unchanged after adjustment for changes in covariates in model 3 (Table 2). Further, more detailed results regarding weight loss are presented in Table 3. The multivariable-adjusted HRs were 0.97 (95% CI, 0.70–1.34) for weight loss of −2.4 to −1.5 kg, 0.98 (95% CI, 0.70–1.38) for −3.4 to −2.5 kg, 1.28 (95% CI, 0.91–1.81) for −4.4 to −3.5 kg, 1.27 (95% CI, 0.92–1.77) for −5.4 to −4.5 kg, and 1.64 (95% CI, 1.29–2.09) for ≤−5.5 kg (Table 3). Additionally, the results of the sensitivity analysis were attenuated modestly after excluding participants who reported an unintentional weight loss of ≤−2 to −3 kg/6 months (model 4). Table 4 shows the results of stratified analyses, but no interaction was observed for each stratified covariate.

**Table 2.** Association between weight change and incident disabling dementia (*n* = 6,672)^a^

	Weight change		

	Weight loss	Stable weight	Weight gain
	(≤−1.5 kg)	(−1.4 to +1.4 kg)	(≥+1.5 kg)
Number of participants	3,113	1,949	1,610
Number of cases	327	141	96
Person years	14,805	9,941	8,119
Incident rate/1,000 person-years	22.1	14.2	11.8
Crude model	1.58 (1.30–1.93)	1.00 (*ref.*)	0.84 (0.65–1.08)
Model 1^b^	1.31 (1.08–1.60)	1.00 (*ref.*)	0.85 (0.66–1.10)
Model 2^c^	1.26 (1.03–1.54)	1.00 (*ref.*)	0.82 (0.63–1.06)
Model 3^d^	1.27 (1.04–1.55)	1.00 (*ref.*)	0.82 (0.63–1.06)
Model 4^e^	1.21 (0.96–1.51)	1.00 (*ref.*)	0.78 (0.59–1.05)

^a^Hazard ratios (HRs) and 95% confidence intervals (95% CIs) were calculated by Cox proportional hazards models.

^b^Model 1 was adjusted for sex and age (continuous).

^c^Model 2 was adjusted for model 1 plus education level (junior high school or lower, high school, college or higher, or missing), smoking status (current, never, former, or missing), time spent walking (<0.5 h, 0.5–1 h, ≥1 h, or missing), psychological distress (yes, no, or missing), history of diseases (stroke, hypertension, myocardial infarction, gastric and duodenal ulcer, diabetes, or cancers[yes, or no]) in 2006, and BMI in 1994 (<18.5 kg/m^2^, 18.5–24.9 kg/m^2^, ≥25.0 kg/m^2^, or missing).

^d^Model 3 was adjusted for model 2 plus changes in daily energy intake (in tertiles, kcal/d), persistently walking for ≥1 h/day (yes, no, or missing), and any new reported diseases (yes, or no) between 1994 and 2006.

^e^Model 4 was adjusted for the same covariates as Model 3 but excluding participants who reported an unintentional weight loss of ≤−2 to −3 kg/6 months at baseline (*n* = 5,448).

**Table 3.** Association between weight change in seven groups and incident disabling dementia (*n* = 6,672)^a^

	Weight change						

	Weight loss					Stable weight	Weight gain

	(≤−5.5 kg)	(−5.4 to −4.5 kg)	(−4.4 to −3.5 kg)	(−3.4 to −2.5 kg)	(−2.4 to −1.5 kg)	(−1.4 to +1.4 kg)	(≥+1.5 kg)
Number of participants	1,052	474	419	529	639	1,949	1,610
Number of cases	138	49	43	45	52	141	96
Person years	4,704	2,240	2,043	2,642	3,176	9,941	8,119
Incident rate/1,000 person-years	29.3	21.9	21.0	17.0	16.4	14.2	11.8
Crude model	2.13 (1.69–2.69)	1.58 (1.14–2.18)	1.50 (1.07–2.12)	1.21 (0.86–1.60)	1.16 (0.65–1.08)	1.00 (*ref.*)	0.84 (0.65–1.08)
Model 1^b^	1.71 (1.35–2.17)	1.28 (0.93–1.78)	1.31 (0.93–1.84)	1.00 (0.73–1.37)	1.00 (0.72–1.41)	1.00 (*ref.*)	0.85 (0.66–1.10)
Model 2^c^	1.62 (1.27–2.07)	1.27 (0.91–1.76)	1.28 (0.90–1.80)	0.98 (0.70–1.37)	0.97 (0.70–1.33)	1.00 (*ref.*)	0.82 (0.63–1.06)
Model 3^d^	1.64 (1.29–2.09)	1.27 (0.92–1.77)	1.28 (0.91–1.81)	0.98 (0.70–1.38)	0.97 (0.70–1.34)	1.00 (*ref.*)	0.82 (0.63–1.06)
Model 4^e^	1.53 (1.15–2.03)	1.19 (0.81–1.74)	1.18 (0.79–1.77)	1.02 (0.71–1.48)	1.01 (0.71–1.43)	1.00 (*ref.*)	0.78 (0.59–1.05)

^a^Hazard ratios (HRs) and 95% confidence intervals (95% CIs) were calculated by Cox proportional hazards models.

^b^Model 1 was adjusted for sex and age (continuous).

^c^Model 2 was adjusted for model 1 plus education level (junior high school or lower, high school, college or higher, or missing), smoking status (current, never, former, or missing), time spent walking (<0.5 h, 0.5–1 h, ≥1 h, or missing), psychological distress (yes, no, or missing), history of diseases (stroke, hypertension, myocardial infarction, gastric and duodenal ulcer, diabetes, or cancers[yes, or no]) in 2006, and BMI in 1994 (<18.5 kg/m^2^, 18.5–24.9 kg/m^2^, ≥25.0 kg/m^2^, or missing).

^d^Model 3 was adjusted for model 2 plus changes in daily energy intake (in tertiles, kcal/d), persistently walking for ≥1 h/day (yes, no, or missing), and any new reported diseases (yes, or no) between 1994 and 2006.

^e^Model 4 was adjusted for the same covariates as Model 3 but excluding participants who reported an unintentional weight loss of ≤−2 to −3 kg/6 months at baseline (*n* = 5,448).

**Table 4.** Association between weight change in seven groups and incident disabling dementia in stratification analysis^a^

	Weight change							*P*-interaction for weight loss^b^

	Weight loss					Stable weight	Weight gain	

	(≤−5.5 kg)	(−5.4 to −4.5 kg)	(−4.4 to −3.5 kg)	(−3.4 to −2.5 kg)	(−2.4 to −1.5 kg)	(−1.4 to +1.4 kg)	(≥+1.5 kg)	
Sex								

Male (*n* = 2,919)								0.213
Model 1^c^	1.72 (1.18–2.49)	1.48 (0.93–2.38)	1.42 (0.82–2.44)	1.27 (0.79–2.04)	0.93 (0.58–1.49)	1.00 (*ref.*)	0.82 (0.56–1.20)	
Female (*n* = 3,753)								
Model 1^c^	1.54 (1.11–2.13)	1.09 (0.69–1.74)	1.21 (0.77–1.91)	0.76 (0.47–1.24)	1.04 (0.67–1.62)	1.00 (*ref.*)	0.83 (0.58–1.19)	

Age in 1994								

<60 years old (*n* = 2,129)								0.216
Model 2^d^	1.94 (0.95–4.00)	1.45 (0.57–3.70)	0.62 (0.14–2.65)	0.73 (0.22–2.50)	1.17 (0.46–2.97)	1.00 (*ref.*)	1.05 (0.54–2.01)	
≥60 years old (*n* = 4,543)								
Model 2^d^	1.83 (1.41–2.37)	1.39 (0.98–1.97)	1.54 (1.08–2.20)	1.13 (0.79–1.60)	1.07 (0.76–1.50)	1.00 (*ref.*)	0.79 (0.59–1.05)	

BMI in 1994								

<25 (*n* = 4,637)								0.601
Model 3^e^	1.78 (1.25–2.52)	1.47 (1.01–2.14)	1.19 (0.78–1.82)	1.06 (0.73–1.56)	0.92 (0.63–1.34)	1.00 (*ref.*)	0.86 (0.64–1.16)	
≥25 (*n* = 1,948)								
Model 3^e^	1.49 (0.92–2.41)	0.90 (0.45–1.82)	1.59 (0.83–3.03)	0.83 (0.38–1.84)	1.24 (0.66–2.35)	1.00 (*ref.*)	0.82 (0.46–1.44)	

^a^Hazard ratios (HRs) and 95% confidence intervals (95% CIs) were calculated by Cox proportional hazards models.

^b^*P*-interaction for weight loss was considered as *P* value of the cross-product term added into the corresponding models.

^c^Model 1 was adjusted for the same covariates in Model 3 in Table 2 except sex.

^d^Model 2 was adjusted for the same covariates in Model 3 in Table 2 except age.

^e^Model 3 was adjusted for the same covariates in Model 3 in Table 2 except BMI in 1994.

## DISCUSSION

We examined the association between long-term weight change since midlife and risk of incident disabling dementia among Japanese elderly. We found that compared with participants with stable weight (−1.4 to +1.4 kg), those with a ≤−3.5 kg weight loss over 12 years showed a higher risk of incident disabling dementia.

Our findings of the association between weight loss and dementia are generally consistent with the results from previous studies,^6^^–^^13^^,^^27^ but most of them were conducted among Caucasians who usually had larger body weight and weight change than Asians. The Whitehall study suggested that a ≤−7.5 kg weight loss over 30 years from midlife to late-life was associated with risk of death from dementia compared to stable weight (−2.5 to +2.5 kg).^7^ While our participants of Japanese elderly were generally with smaller weight, and a relatively smaller weight loss (ie, ≤−3.5 kg) over 12 years seemed to be associated with an elevated dementia risk.

Short-term weight loss within about 6 months to 1 year was used to evaluate frailty among the elderly,^25^^,^^26^ which has been indicated as a predictor of dementia.^28^ We conducted a sensitivity analysis where excluding people who reported a ≤−2 to −3 kg weight loss during the past 6 months, which was one indicator of frailty; however, the results were modestly attenuated suggesting that frailty could partly explain the association of weight loss with dementia but that other pathologies could be possible. For example, cardiovascular risk factors play an important role in dementia development. A previous study showed that compared to American adults with stable weight, those with a >3% weight loss were associated with elevated risk of stroke, which was a major factor for vascular dementia and Alzheimer’s disease (AD).^29^ Moreover, weight loss and the associated alterations in adipose tissue function and structure could also negatively affect brain health.^10^ Also, weight loss could be caused by pre-existing type 2 diabetes, which is a risk factor for dementia. A previous study showed that weight loss among diabetes patients was associated with an increased risk of dementia.^30^ Additionally, it was reported that weight loss might be directly associated with changes in brain structure,^31^^–^^33^ and one study suggested that the association between weight loss, AD biomarkers and brain atrophy in healthy participants remained significant even after the exclusion of subjects with progression to mild cognitive impairment or dementia in the follow-up.^33^

Japanese adults may start to lose weight in late midlife because of less energy intake and physical activity,^34^ but it remains unknown whether weight loss starting from midlife would also be related to dementia among Japanese. We analyzed the data stratified by age in 1994, (<60 or ≥60 years) and observed that a ≤−4.5 kg weight loss among younger individuals (ie, <60 years in 1994) was associated with an elevated disabling dementia risk. Similarly, previous studies with longer follow-up also found that weight loss over 5 to 30 years among an even younger population was associated with higher risk of dementia.^7^^–^^9^^,^^35^

Nevertheless, we could not rule out the possibility of reverse causality where weight loss might act as a marker of dementia development and start as early as 1 or 2 decades before diagnosis.^33^^,^^36^ Prior studies explained that impairment in smelling, swallowing, or chewing functions during the preclinical phase of dementia may contribute to decreased appetite, reduced energy intake, and consequent weight loss.^37^^,^^38^

Our study had some limitations. First, although the response rate was relatively high (72.9%), we cannot rule out the possibility of selection bias among the current study population. Unfortunately, we have no information regarding non-responders. Second, body weight was self-reported, and therefore may have been underestimated by participants, and it is unclear to what extent weight change in the present study reflected objective measurements. Third, we did not ask participants about their reason for losing weight. Weight loss could be unintentionally caused by chronic diseases or could be an intentional decision. Despite these limitations, our study had some strengths, including the large number of participants, the high rate of follow-up and considerable control of confounding factors.

In conclusion, our study suggested that a ≤−3.5 kg weight loss over 12 years was associated with a higher risk of incident disabling dementia among elderly Japanese. An unintentional weight loss within a short period is easy to notice, but a long-term one is more often to be neglected. Thus, the present finding is a useful message to geriatricians, that long-term weight loss also should be taken seriously and that regular weight check since midlife may serve as a cost-effective tool to screen those at-risk of dementia.
